# Localized Breast Amyloidosis Associated with Sjörgren Syndrome

**DOI:** 10.1155/2020/8828263

**Published:** 2020-06-24

**Authors:** Edgar G. Fischer

**Affiliations:** Division of Surgical Pathology and Cytopathology, Department of Pathology MSC08 4640, University of New Mexico, Albuquerque, NM 87131, USA

## Abstract

Sjörgren syndrome is a systemic autoimmune disease that is rarely associated with amyloid deposits, and in most reported cases, these deposits are localized to a single organ. Amyloidosis of the breast is a rare and unexpected finding, and only 5 case series with 63 patients have been published in the past 40 years. To date, only 6 cases have been reported in which Sjörgren syndrome is associated with amyloid deposits in the breast. A 61-year-old female diagnosed with Sjörgren syndrome underwent a breast needle core biopsy for calcifications. Microscopic examination revealed amyloid deposits in the periductular basement membranes, in the walls of arteries and veins, and in the surrounding connective tissue. No malignancy was found. Clinical workup revealed the amyloid deposits to be localized to the breast and did not reveal an underlying hematolymphoid neoplasm. The association between Sjörgren syndrome and breast amyloidosis is rare, but few reports have appeared in recent years, and it may be an emerging disease association. The finding of localized amyloid in the breast and other organs should lead to a clinical workup not only for hematopoietic neoplasms but also for autoimmune diseases such as Sjörgren syndrome.

## 1. Introduction

Sjörgren syndrome is an uncommon systemic autoimmune disease characterized by dry eyes (keratoconjunctivitis sicca) and dry month (xerostomia) [1]. The disease results from an autoimmune process of unknown etiology that is directed against lacrimal and salivary glands. A recent review of the literature found Sjörgren syndrome associated with amyloidosis in 57 cases [2]. Amyloid deposits were localized in the majority of cases, mainly to the skin and lung. They were systemic in only 3 cases, and only 2 patients had amyloid deposits in the breast [2]. Sjörgren syndrome is associated with B-cell hyperactivity, and patients have an increased risk of hematolymphoid disorders, specifically marginal zone B-cell lymphomas [2, 3].

Amyloidosis is a heterogeneous group of diseases defined by deposits of abnormal extracellular fibrillary proteins that cause tissue damage. The most common types are AL and AA amyloidosis. In AL amyloidosis, deposits are composed of immunoglobulin light chains secreted in hematolymphoid disorders such as plasma cell dyscrasia or multiple myeloma. In AA amyloidosis, deposits are derived from serum amyloid A- (SAA-) associated protein. SAA protein production by the liver is increased in chronic inflammatory conditions, including autoimmune disorders such as rheumatoid arthritis, and in chronic infections. Amyloidosis of the breast was first described in 1973 [4]. It is a rare diagnosis and can occur as localized disease without extramammary involvement or as part of systemic amyloidosis. Five series of 3 or more breast amyloidosis cases have been published in the past 40 years and comprise a mixture of cases with either localized breast or systemic involvement [5–9]. The association of Sjörgren syndrome with localized breast amyloidosis is rare. It appears to be an emerging association, with 6 cases reported to date. The current case is the seventh case reported in which breast amyloidosis is associated with Sjörgren syndrome.

## 2. Case Presentation

A 61-year-old female presented with calcifications of the left breast on mammography and underwent stereotactic vacuum-assisted core biopsy. The patient had a history of Sjörgren syndrome, chronic renal failure, hypertension, anemia, lymphadenopathy, arthritis, rash, restrictive pulmonary disease, and gastrointestinal symptoms. Clinical workup with a bone marrow biopsy, lymph node biopsy, and splenectomy did not show evidence of a hematolymphoid neoplasm or systemic amyloidosis, and a small bowel biopsy was unremarkable. Her chronic renal failure was attributed to hypertensive renal disease. Six years after her breast biopsy, she developed end-stage kidney disease and entered hospice care.

**Figure 1:**
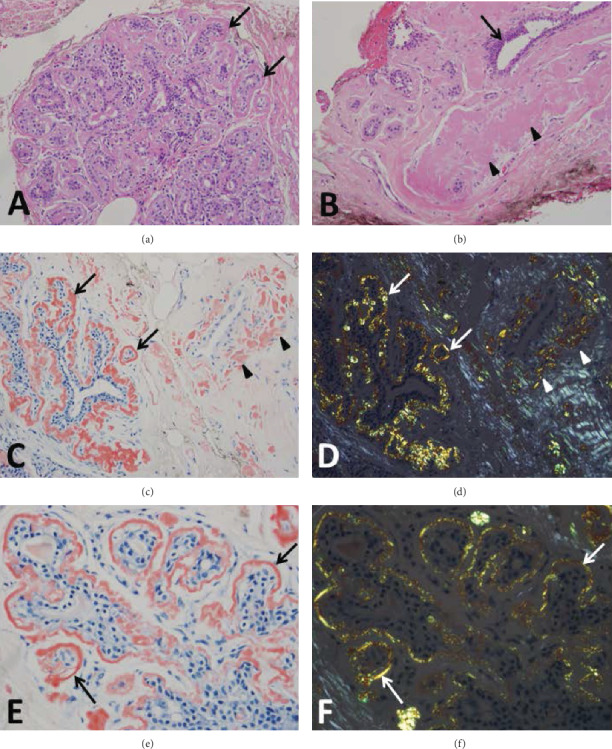
(a) A breast lobule shows periductular basement membranes (arrows) thickened by eosinophilic amyloid deposits (H&E, 200x). (b) An atrophic breast lobule and a duct (arrow) are surrounded by eosinophilic amyloid deposits (arrowheads) (H&E, 200x). (c) Congophilic amyloid deposits in periductular basement membranes (arrows) and in perivenular interstitium (arrowheads) (Congo red, 200x). (d) Corresponding image under polarized light shows apple green birefringence of periductular (arrows) and perivenular (arrowheads) amyloid deposits as well as gray-white birefringence of interstitial collagen (Congo red, 200x). (e) A breast lobule with amyloid deposits in periductular basement membranes (arrows) (Congo red, 400x). (f) Corresponding image with apple green birefringence (arrows) under polarized light (Congo red, 400x).

H&E-stained sections of the 9-gauge vacuum-assisted breast core biopsy showed benign breast parenchyma with amyloid deposits in various stages (Figures 1(a) and 1(b)). Multiple breast lobules had marked thickening of periductular basement membranes by eosinophilic deposits (Figure 1(b)). Ductules surrounded by heavy deposits appeared atrophic or partially replaced by amyloid (Figure 1(b)). Deposits were also present in the periductal and perivascular interstitial areas and in the walls of arteries and veins (Figures 1(c) and 1(d)). The Congo red stain highlighted more subtle amyloid deposits in periductular basement membranes (Figure 1(e)). Congophilic deposits demonstrated apple green birefringence under polarized light, while areas of collagenous stroma had gray-white birefringence (Figures 1(d) and 1(f)). No epithelial atypia, neoplasm, or hematolymphoid lesion was present. Given the negative clinical workup, the amyloid deposits in the breast were considered localized and not associated with systemic amyloidosis.

## 3. Discussion

This report describes only the seventh case of Sjörgren syndrome associated with breast amyloidosis. Amyloid deposits were localized to the breast. This is the fourth case reported in 2019 and 2020, suggesting that this may be an emerging association. A recent comprehensive review of Sjörgren syndrome associated with amyloidosis reported 57 cases, and the vast majority of patients had localized deposits, most often in the skin and lung [2]. Sjörgren syndrome patients rarely have systemic amyloidosis, and only 3 of 57 cases had systemic deposits. Only two patients in this review had amyloid deposits in the breast, including one with a primary breast marginal zone lymphoma (Table 1) [2]. Sjörgren syndrome is uncommon, with an estimated incidence of 3-11 per 100,000 [1]. Although it is associated with an increased risk of lymphoma [1], only one of the reported cases with breast amyloidosis had a documented hematolymphoid malignancy, a marginal B-cell lymphoma that was also located in the breast (Table 1) [10].

Breast amyloidosis is a rare diagnosis. Only 5 case series with a total of 63 cases have been published in the past 40 years (Table 2) [5–9]. Only 2 of these 63 patients also had Sjörgren syndrome (Table 2). Five cases with this association have been published since 2002 (Table 1). Three of these 5 cases were reported in 2019 and 2020, and the association between these 2 diseases may be increasingly recognized.

Of the 57 cases summarized by Hernandez-Molina et al., most had AL-type deposits, and only 7 (12%) patients had an associated lymphoma [2]. This is in contrast to rheumatoid arthritis and ankylosing spondylitis, where most deposits are of the AA type [2]. These authors suggest that amyloid deposits in Sjörgren syndrome may be secondary to hypergammaglobulinemia related to B-cell hyperactivity rather than secretion of monoclonal light chains by a hematolymphoid neoplasm [2].

As surgical pathologists examine breast biopsies, unexpected abnormalities such as amyloid deposits are rarely encountered. Breast amyloidosis has been associated with other autoimmune disorders that include systemic lupus erythematosus, rheumatoid arthritis, and polymyalgia rheumatica [7]. Their clinical significance lies in the possible association with systemic amyloidosis, plasma cell neoplasms, lymphomas, and systemic inflammatory diseases and should trigger an appropriate clinical workup.

## Figures and Tables

**Table 1:** Reported cases of breast amyloidosis associated with Sjörgren syndrome. All patients were female.

Age (years)	Laterality	Breast clinical findings	Associated condition	Amyloid type	Year published	Reference
60	Bilateral	Induration	Left breast amyloid associated with invasive carcinoma		2002	[8]
37	Left	Microcalcifications	Marginal B-cell lymphoma	AL	2003	[10] reference in [2]
63		Microcalcifications		AL	2006	[11] reference in [2]
67		Calcifications			2019	[7]
45	Right	Palpable lump, normal mammogram			2019	[12]
41					2020	[13]
61	Left	Calcifications				Current case

**Table 2:** Case series of breast amyloidosis published in the past 40 years.

No. of cases	Cases with Sjörgren syndrome	Comments	Year published	Reference
3	1		2002	[8]
7	0	Amyloid localized in all cases, none had plasma cell dyscrasia	2011	[5]
40	0		2013	[9]
3	0		2015	[6]
10	1	Amyloid localized in all cases	2019	[7]

## Data Availability

No other data or other supporting materials were collected or used for this report.
